# Hepatitis E Virus Genotype 3 among Hemodialysis Patients in Mexico: First Identification of Chronic Infection

**DOI:** 10.3390/pathogens13070578

**Published:** 2024-07-11

**Authors:** Edgar D. Copado-Villagrana, Ilsy X. Duarte-López, Arturo Calderón-Flores, Isidro Loera-Robles, Oliver Viera-Segura, Nora A. Fierro

**Affiliations:** 1 Unidad de Medicina Familiar 5, Instituto Mexicano del Seguro Social, Nogales 84000, Mexico; edcovi92@gmail.com (E.D.C.-V.);; 2 Departamento de Inmunología, Instituto de Investigaciones Biomédicas, Universidad Nacional Autónoma de México, Mexico City 04510, Mexico; 3 Instituto en Investigación en Ciencias Biomédicas, Centro Universitario de Ciencias de la Salud, Universidad de Guadalajara, Guadalajara 44340, Mexico; o.vierasegura@gmail.com

**Keywords:** viral hepatitis, extrahepatic manifestations and hepatitis E, risk factors and hepatitis E, HEV strains

## Abstract

The global distribution of hepatitis E virus (HEV) is attributed to its capacity to spread through several routes of transmission; hemodialysis has gained increased amounts of attention in recent years. Although Mexico is considered a hyperendemic region for hepatitis E, no HEV surveillance is performed in the country. The frequency of HEV in hemodialysis (HD) patients has not been determined. Herein, we conducted a cross-sectional single-center analytical study including 67 serum samples from HD patients. Anti-HEV IgG and IgM antibodies and the viral genome were determined; partial regions within the HEV genome were sequenced for further phylogenetic analysis. Globally, 14.9% of the tested patients exhibited reactivity for IgG antibodies against HEV, and none showed reactivity to IgM. A total of 5.9% of the samples showed HEV genome amplification, and sequencing confirmed the identity of genotype 3; subsequent analysis of positive cases revealed two acute cases and chronic hepatitis E infection in one patient. Notably, the chronic patient was negative for anti-HEV IgG antibodies. Our findings highlight the importance of viral genome testing in HD patients and the need to establish guidelines for HEV detection in Mexico.

## 1. Introduction

Hepatitis E is an infection caused by the hepatitis E virus (HEV); this virus is the main etiology of acute hepatitis worldwide [1]. The World Health Organization (WHO) estimates that more than one-third of the population is exposed to HEV, suggesting that it is an agent of global relevance [2]. This is especially important considering that despite being initially thought to be a benign and self-limiting disease, chronic hepatitis E, determined by the presence of viral RNA for more than three months [3] and leading to cirrhosis, is frequent in patients with immunosuppression. Hepatitis E can also lead to severe illness in specific populations, and the adverse effects of HEV in diverse tissues [4] support the notion of the systemic nature of the disease. Therefore, characterizing risk groups for contracting and/or transmitting the infection is a priority.

HEV is a single-stranded, positive-sense RNA virus of approximately 7.2 kb with 3 to 4 open reading frames (ORFs) encoding nonstructural and structural proteins. This virus belongs to the genus *Paslahepevirus* within the *Hepeviridae* family. The species *Paslahepevirus balayani* affects humans and comprises eight genotypes (gt); two genotypes, genotype 1 (gt1) and genotype 2 (gt2), are restricted to infecting humans, while genotypes 3 (gt3), 4 (gt4), and 7 (gt7) have exhibited zoonotic abilities by infecting both humans and diverse animal species [5,6]. Zoonosis via rodent HEV, a *Rocahepevirus ratti* species, has recently been demonstrated [7].

The gt1 and gt2 genotypes are predominantly associated with waterborne transmission in developing countries, while gt3 and gt4 are commonly zoonotically transmitted in industrialized regions. In addition to zoonosis and the fecal–oral route, which are considered the main transmission routes of the virus, the transfusion of blood products and hemodialysis are other potential transmission sources [8,9].

Although, to date, no cases of chronic illness associated with hemodialysis (HD) patients have been reported, a relationship between hepatitis E and kidney dysfunction commonly linked to gt3 has been documented [10]. Moreover, considering that HD patients are in a relative state of immunosuppression [11] and that some of them are potential kidney transplant recipients, there is a notable risk for the development of severe chronic hepatitis E in these patients; consequently, they should be considered a high-risk group for infection. In this regard, the seroprevalence of HEV among HD patients across the world has been investigated, reporting a range of frequencies from 0% to 44% [9]. Furthermore, some studies have shown greater circulation of the virus among HD patients than in the general population [9,12,13]. However, data related to viremia and HEV genotypes in HD patients are still limited. This is especially important in HEV-endemic countries.

Since two outbreaks in Mexico in the late 1980s, whose sampling resulted in the first identification of gt2 in the world, the country is considered a hyperendemic region for hepatitis E [14]. However, we still lack a routine diagnosis for the infection, and the Ministry of Health in Mexico does not report the epidemiological status of the infection in the country. Therefore, hepatitis E is still a neglected disease in Mexico, and information related to this disease comes from independent research groups. Recently, we reported the circulation of gt1 [15] and gt3 [16] in humans. Previous studies confirmed the circulation of gt3 in swine [17,18], suggesting a potential risk of zoonosis in the country. Moreover, we identified HEV RNA among blood donors [19]. In addition, serologic studies in the country have reported the presence of anti-HEV antibodies, which reach frequencies as high as 58% in obese patients with liver disease [14]; this indicates the wide circulation of this virus in Mexico and the need to follow up hepatitis E in potential risk groups, including HD patients.

Herein, we aimed to detect anti-HEV (IgG and IgM) antibodies along with viral RNA and perform phylogenetic analysis of positive cases of infection among patients undergoing hemodialysis in a hospital located in a city on the northwestern border of Mexico.

## 2. Materials and Methods

### 2.1. Study Population

In this cross-sectional analytical study, 67 blood samples from HD patients from the Hospital General de Zona No. 5 (HGZ 5) of the Instituto Mexicano del Seguro Social (IMSS), located in the City of Nogales, Sonora, in northwest Mexico, were included. The inclusion criteria for participants were as follows: were patients of any age and sex at any stage of the disease, regardless of the time they had been receiving hemodialysis; were residents of any municipality in the northwestern region of Mexico who attended hemodialysis sessions at HGZ5; and agreed to participate in the study and signed a letter of informed consent. Participants were recruited between 1 February and 28 February 2023. Chronic hepatitis E was assessed by detecting viral RNA for over three months, confirmed with a second sample from patients who tested positive for the HEV genome in the initial analysis. After participants provided informed consent, blood samples were obtained by venipuncture, and the serum was immediately recovered by centrifugation. The samples were aliquoted and stored at −70 °C until use. The experimental study was conducted at the Laboratorio de Inmunología, Instituto de Investigaciones Biomédicas, Universidad Nacional Autónoma de México.

### 2.2. Clinical and Demographic Data

The clinical, epidemiological, and demographic information was collected using a structured questionnaire. The recorded data included age, sex, demographic information, clinical features, and risk factors for HEV infection. The municipality of residence, occupation, work with animals, type of catheter for hemodialysis, time on hemodialysis, transfusions, contact with patients with liver disease, housing, excreta management, pets, and consumption of pork or derivatives were recorded.

### 2.3. Serological Tests

The serum samples were tested individually for the presence of anti-HEV immunoglobulin M (IgM) and immunoglobulin G (IgG) antibodies by using the commercial enzyme-linked immunosorbent assay (ELISA) MIKROGEN DIAGNOSTIK kits (Neuried, Germany) recomWell HEV IgG and recomWell HEV IgM following the manufacturer’s instructions. Briefly, 10 µL of serum from patients diluted in 100 µL of dilution buffer (provided with the ELISA kit) was deposited in wells coated with recombinant HEV antigen. After an incubation period of 1 h and four washing cycles, a conjugate of anti-human IgG or IgM and peroxidase was added. After 30 min of incubation and four washing cycles, peroxidase substrate was added to start the reaction, which was stopped after 30 min. Finally, the absorbance of each well was read in a WHYM201 microplate reader (Poweam Medical Co., Ltd., Nanjing, China). The cutoff value was calculated based on the absorbance results of the kit controls; all patients with values above this cutoff were considered reactive.

### 2.4. HEV RNA Extraction and RT–Semi-Nested PCR

All the samples were analyzed individually for the determination of viral RNA. Initially, RNA extraction was performed using the QIAamp Viral RNA Mini Kit (QIAGEN, Germantown, MD, USA), and modifications were made in accordance with the manufacturer’s instructions. For each serum sample, 1200 µL of Buffer AVL-RNA carrier was mixed thoroughly with 300 µL of serum, and after 10 min of incubation at room temperature, 1200 µL of absolute ethanol was added to the solution and mixed. Then, 630 µL of the lysate was applied to a QIAamp Mini column and centrifuged. This procedure was repeated until all the lysate was loaded onto the column. Washing buffers were added according to the manufacturer’s specifications, and RNA was eluted with 35 µL of nuclease-free water. RNA aliquots (5 µL) were immediately used for reverse transcription (RT) reactions or stored at −80 °C until use.

HEV genome screening was performed by targeting the conserved ORF-2/3 overlapping region with the primers described by Inoue et al. [20]. The first-round PCR primers used were HE363 and HE361, which produced a PCR product of 161 bp (nucleotide positions 5302 to 5461 according to GenBank AB089824) [20]. The second semi-nested PCR primers used were HE363 and HE366, which produced a 137 bp PCR product (nucleotide positions 5325 to 5461 according to GenBank AB089824) [20]. RT–PCR was carried out using an Invitrogen SuperScript III One-Step RT–PCR System with a Platinum Taq DNA Polymerase System (Thermo Fisher Scientific, Waltham, MA, USA).

For RT–PCR and first-round PCR, 5 µL of RNA, 10 pmol of (F) HE361 primer (5′-GCRGTGGTTTCTGGGGTGAC-3), and 10 pmol of (R) HE363 primer (5′-GMYTGGTCDCGCCAAGHGGA-3) were mixed and incubated at 70 °C for 5 min; subsequently, the tubes were transferred to an ice bath for 5 min, after which a master mixture containing 10 µL of 2X of concentration of SuperScript III One-step RT–PCR mixture, 0.3 µL of the enzyme mixture, and nuclease-free water was added to a final volume of 20 µL per tube. One hour of incubation at 50 °C for RT was followed by 5 min of incubation at 90 °C to inactivate reverse transcriptase and then a PCR program consisting of initial denaturation at 94 °C for 5 min, 35 cycles of 94 °C for 30 s, 60 °C for 30 s, 72 °C for 30 s, and a final extension at 72 °C for 5 min [20]. Semi-nested second-round PCR was performed with 2 µL of the first round of PCR containing 10 pmol of the HE366 primer (5′GYTGATCTCAGCCCTTCGC-3′), 10 pmol of the HE363 primer, 10 µL of 2X reaction mix, 0.3 µL of enzyme and nuclease-free water to a final volume of 20 µL per tube. The same PCR protocol was used for the first and second rounds of PCR. The presence of the PCR products was analyzed using 1.5% agarose gel electrophoresis. Gels were stained with SmartGlow PS stain (Accuris, Edison, NJ, USA) and visualized with a UV transilluminator; positive samples showed a 137 bp DNA band [16,20].

To determine the HEV genotype in the samples positive for the presence of viral RNA, the methyltransferase region within ORF1 was amplified with primers described by Wang et al. [21]. The primers ConsORF1-S1, ConsORF1-S-2, and ConsORF1-A2 corresponding to nucleotide positions 56 to 79 bp, 104 to 124, and 389 to 367 bp, respectively, according to the Burmese isolated GenBank M72318 were used [21]. RT and first-round PCR were performed by adding 10 pmol of each of the ConsORF1-S1 (5′-CTGGCATYACTACTGCYATTGAGC-3′) and ConsORF1-A2 (5′-GGCAGWRTACCARCGCTGAACATC-3′) primers to 5 µL of RNA. The mixture was incubated for 5 min at 70 °C and transferred to the ice for 5 min, after which a master mixture containing 10 µL of 2X SuperScript III One-step RT–PCR mixture, 0.3 µL of the enzyme mixture, and nuclease-free water was added to a final volume of 20 µL per tube. The RT program consisted of 20 min at 42 °C, 40 min at 50 °C, and 5 min at 90 °C, followed by a PCR program consisting of a denaturation step at 94 °C for 5 min; 40 cycles of 94 °C for 45 s and 45 °C for 45 s; 68 °C for 30 s; and a final extension at 68 °C for 5 min [16]. For the second round of semi-nested PCR, 2 µL of the first PCR was mixed with 10 pmol of ConsORF1-S2 primer (5′-CTGCCYTKGCGAATGCTGTGG-3′), 10 pmol of HEA-2 primer, 10 µL of the reaction mix, 0.3 µL of SuperScript III One-step RT–PCR enzyme and water to a final volume of 20 µL. The PCR program was the same as that for the first round of PCR. Agarose electrophoresis was performed to analyze the presence of a 285 bp DNA band [16,21].

To distinguish acute and chronic infections in those individuals for whom the HEV genome was initially detected, the presence of viral RNA for more than three months was tested in a second sample following a previously described procedure.

### 2.5. Partial ORF-1 Sequencing and Phylogenetic Analysis

The PCR products from ORF-1 were purified from agarose gels with a Wizard^®^ SV Gel and PCR Clean-Up System (Promega, Woods Hollow Road, Madison, WI, USA) and sequenced by an external provider. Nucleotide sequences obtained from four samples were aligned with the GenBank HEV genotype references proposed by Smith et al. [22] using MAFFT v.7.525. The region comprising homologous nucleotides 138 to 377 of the consensus alignment with AF082843 was analyzed via MrBayes 3.2.7 for phylogenetic Bayesian inference [23]. The evolutionary model used was General Times reversible with gamma-distributed rate variation across sites and a proportion of invariable sites; analysis was run using Markov chain Monte Carlo simulation for 300,000 generations.

### 2.6. Statistical Analyses

The qualitative variables are presented as absolute and relative frequencies, while the quantitative variables as medians and interquartile ranges (IQRs). All the statistical comparisons were performed using SPSS software version 25 (IBM, Inc., Armonk, NY, USA). Based on the normality test results (Kolmogorov–Smirnov) and the distribution characteristics of the quantitative variables, comparisons of patient populations with positive and negative RT–PCR results were carried out using the Mann–Whitney U test. Fisher’s exact test was used for qualitative variables, considering the population’s characteristics. A *p* value less than 0.05 was considered to indicate statistical significance.

## 3. Results

### 3.1. IgG Anti-HEV Antibodies in HD Patients

Overall, the median age in our cohort was 52 years; housing in urban areas was common for most of the included patients, who were mostly male (Table 1). Of the 67 samples tested, 14.9% were positive for IgG antibodies against HEV, and none showed reactivity to IgM (Table 2).

The groups of patients who were reactive and nonreactive to IgG were compared, but no statistically significant differences were found despite the observation of higher proportions of certain variables. For example, 70% of the reactive patients were older than 50 years, 60% were female, 80% had a temporary catheter for hemodialysis, and 90% had been receiving hemodialysis for less than three years.

### 3.2. Acute and Chronic Hepatitis E Infections Were Identified in HD Patients

Four of the sixty-seven samples were positive for the presence of HEV RNA. Of the positive samples for the viral genome, one was also positive for IgG anti-HEV (Table 2). Importantly, subsequent follow-up of more than three months of patients with prior detection of viral RNA revealed that the viral genome was still detected in one patient, indicating chronic infection; the initial and second samples recovered from this patient were negative for IgG anti-HEV. The second patient who underwent initial HEV RNA detection and was negative for antibodies died before the second sampling was conducted, and the remaining two patients who were positive for the HEV genome in the initial sampling were negative for HEV RNA in the second testing, indicating that acute and self-limiting infection was identified in the initial analysis. However, no IgM anti-HEV antibodies were detected, whereas the analysis of the follow-up samples from these two patients revealed that one patient was positive for IgG anti-HEV and the other was negative.

### 3.3. Sequencing and Phylogenetic Analysis of HEV-ORF-1 Revealed HEV-gt3 in HD Patients

**Figure 1:**
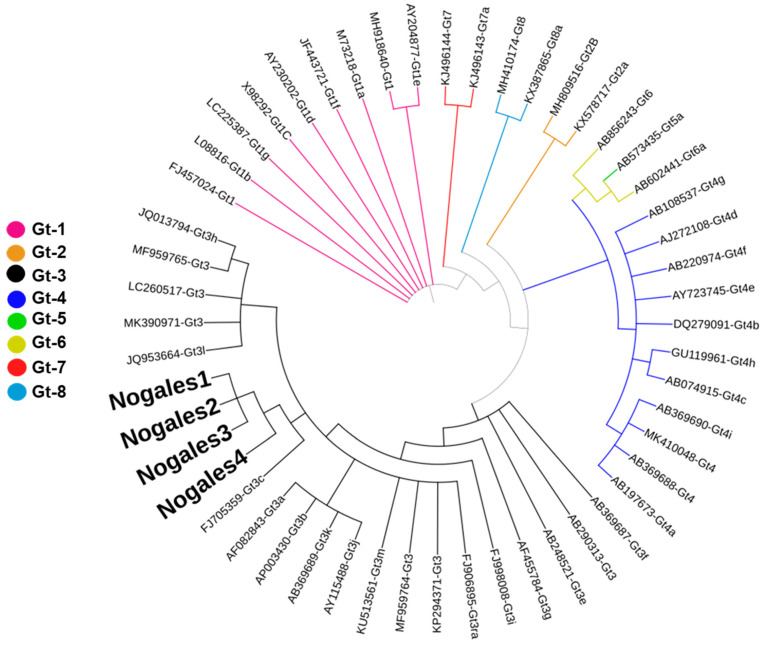
Phylogenetic analysis of ORF-1 HEV sequences from Mexico and HEV reference sequences [23]. The tree was generated via Bayesian inference analysis, and branches of the different genotypes exhibited high credibility values. The sequences obtained in this study were clustered together with reference sequences of HEV genotype 3.

RT–PCR products from positive samples corresponding to the HEV ORF-1 (138 to 377 bp according to AF082843) region were sequenced via the Sanger method and submitted to GenBank (accession numbers: PP209107-PP209110). The sequences obtained in this study and labeled Nogales 1, 2, 3, and 4 were aligned with MAFFT to reference HEV sequences from all the genotypes and analyzed via the MrBayes program for phylogenetic inference. The resulting phylogenetic analysis showed a 0.015 standard deviation in the split frequencies after 300,000 generations, suggesting statistical convergence of the resulting tree (Figure 1); the Nogales sequences clustered closely with the HEV gt3 reference sequences, underscoring that the infecting HEV genotype was gt3.

## 4. Discussion

Hemodialysis has long been recognized as an important risk factor for viral hepatitis, as underscored by the finding that the risk of HCV infection increases with the duration of exposure to this procedure [24]. Similarly, the prevalence of HBV and HCV is greater in HD patients than in the rest of the population [25]. However, information on HEV in the hemodialysis setting is still limited; this is important given that, unlike other viruses that affect the liver and whose endemicity is restricted to specific geographic regions, the circulation of HEV is nearly complete worldwide [26]. Herein, for the first time, we reported acute infections and a case of chronic hepatitis E in the absence of IgG anti-HEV antibodies among HD patients from Mexico.

The frequency of IgG anti-HEV antibodies we found (14.9%) in the total cohort was greater than the 10% reported in Argentinian HD patients [27], whose study showed that the IgG anti-HEV seroprevalence was greater in this group than in healthy controls (4.3%) [28], supporting the notion that HEV could be transmitted through hemodialysis sessions or by iatrogenesis during patient health care [29].

Several studies have compared the seroprevalence of HEV in HD patients with that in the general population. In 2015, Scotto et al. reported a seroprevalence in the general population of 3.7% in 801 subjects and 6% in 231 HD patients in Italy [30]. A study by Stefanidis et al. reported a seroprevalence of 4.8% in a sample of 351 patients from various hemodialysis units in Greece. This indicates that this rate is greater than that in the general population, with a seroprevalence of 0.26% [12]. Similarly, Zekavat et al. reported a seroprevalence of 6.3% in 80 patients on hemodialysis and 2.98% in 276 healthy subjects from southwestern Iran [13]. Herein, the prevalence of anti-HEV antibodies in HD patients was not compared with that in the general population, as this is the first study to address the problem of HEV infection relative to that of hemodialysis in a geographic region, specifically in northwest Mexico, close to regions previously identified with a high HEV seroprevalence. However, we recently reported a seroprevalence of 9.8% for HEV in blood donors from Jalisco, located in western Mexico [19]; that is, a difference of five percentage points less than what we reported in our group of HD patients, suggesting that patients on HD have a greater risk of acquiring the infection than the rest of the population. Furthermore, Tavakoli’s meta-analysis reported the highest HEV seroprevalence in patients with more than five years of exposure to hemodialysis [31]. In our study, patients had been on hemodialysis for less than three years. Thus, to effectively determine the risk of infection, it will be essential to develop prospective studies in which consecutive screenings are carried out on all patients newly admitted to hemodialysis and follow up each patient for long periods. Moreover, screening for HEV infection prior to hemodialysis sessions will allow us to delineate the risk of this procedure for infection.

As can be inferred from the distinct frequencies of HEV among HD patients in diverse countries, its prevalence depends on its circulation among the general population. Our finding of a high incidence of HEV in a city bordering the USA is particularly relevant because of the continuous flow of people between Mexico and its northern neighbors. Indeed, the USA is one of the countries in the Americas with the highest seroprevalence of HEV, with a mean of nine percent [32]. Considering the high seroprevalence of HEV, we found in this study that joint efforts of health systems are needed to address the relevance of HEV epidemiology in Mexico and prevent its dissemination. Currently, legislation in Mexico requires screening for HBV, HCV, and HIV in patients recently admitted to hemodialysis. However, screening for HEV is not mandatory in the country [33].

Our finding of viremia in HD patients is relevant because of the low or even null frequency of viremic cases commonly reported in these patients worldwide [34,35]; for instance, from a study of 231 HD patients in Italy, only 1.2% of cases positive for viral RNA were reported to be gt1 and gt3, the genotypes identified. We detected viremia in 5.9% of the patients studied, and gt3 was present. Even more relevant is our finding of chronic infection in one patient where gt3 was identified. This finding is consistent with the fact that chronic HEV infection is mostly associated with this genotype. Moreover, the absence of IgG anti-HEV antibodies in this patient could be related to the relative immunosuppression status of HD patients since it has been shown that the reliability of antibody tests is reduced in this population [26]. In this regard, considering that HD patients are potential kidney transplant recipients, they may be prone to developing more adverse effects resulting from chronic hepatitis E. Moreover, a potential immunosuppressive status in HD patients will also explain the lack of detection of IgM anti-HEV antibodies in the analyzed cohort, including those cases where acute infection was identified, and suggest that serologic tests are disadvantageous for identifying recently acquired infections and even chronic infections during hemodialysis.

In our study, biochemical data on liver function were not collected during the initial sampling, and no evidence of liver disease was found in the follow-up samples corresponding to acute infection. Importantly, even though no altered transaminase or bilirubin levels were detected in the case of chronic hepatitis E three months after the initial screening, this patient continued to be followed up. Most HEV cases spontaneously resolve the infection in immunocompetent settings, so the treatment is symptomatic. If serious data are identified, the administration of ribavirin is recommended for patients in transplant protocols with HEV RNA detection; close monitoring is needed, and to the extent possible, immunosuppressive treatment should be started after the infection has subsided, with the intention of reducing the risk of chronic disease development [10]. All these measures have been implemented mainly in Europe and Asia. Unfortunately, we still lack specific guidelines for Latin America. Therefore, the data presented reveal the urgent need to establish precise guidelines for case management in endemic regions.

Finally, considering that renal manifestations have been increasing in HEV-infected patients in recent decades, irrespective of acute or chronic hepatitis E [10,36], the possibility of an effect of the virus on kidney function leading to the need for hemodialysis cannot be ruled out. Therefore, future prospective studies are needed to evaluate this possibility.

Our findings highlight the urgency of incorporating HEV into epidemiological surveillance systems to obtain real case statistics, prevent its transmission in Mexico, and emphasize the importance of studying vulnerable populations in endemic regions. All patients on hemodialysis should undergo scrutiny using molecular tests to identify HEV RNA; this is particularly important considering our data showing the inability of serological tools to follow infection in the hemodialysis setting. Furthermore, identifying the viral genome is crucial for identifying chronic HEV infection, whereas monitoring circulating strains is important for identifying the sources of the virus and for the clinical management of patients. Our data are expected to enable clinicians to better handle hepatitis E.

## Figures and Tables

**Table 1:** Clinical and sociodemographic features of the cohort.

Characteristic	N: 67
Median age in years (IQR ^1^)	52 (21.0)
Male (%)	40 (60)
Residence in Nogales (%)	59 (88)
Currently working (%)	15 (22)
Work with animals (%)	10 (15)
Arteriovenous fistula (%)	27 (40)
Median years receiving hemodialysis (IQR)	2 (3.1)
Transfusions (%)	39 (58)
Contact of patients with liver disease (%)	3 (4)
Rural housing (%)	3 (4)
Drainage for excreta management (%)	67 (100)
Pets at home (%)	31 (46)
Consumption of pork or derivatives (%)	49 (73)
Food consumption outside the home (%)	41 (61)

^1^ Interquartile range.

**Table 2:** Overview of the serological results and viral genome detection among HD patients.

**Characteristic**	**N: 67**
Reactive to IgM anti-HEV (%)	0 (0)
Reactive to IgG anti-HEV (%)	10 (14.9)
Positive RT–PCR (%)	4 (5.9)
Positive RT–PCR (N: 4)/Reactive to IgG anti-HEV (%)	1 (25)

## Data Availability

Viral RNA-positive samples were sequenced and submitted to GenBank (accession numbers: PP209107-PP209110).
